# Large scale expression and purification of secreted mouse hephaestin

**DOI:** 10.1371/journal.pone.0184366

**Published:** 2017-09-07

**Authors:** Chandrika N. Deshpande, Vicky Xin, Yan Lu, Tom Savage, Gregory J. Anderson, Mika Jormakka

**Affiliations:** 1 Structural Biology Program, Centenary Institute, Sydney, New South Wales, Australia; 2 Sydney Medical School, University of Sydney, Sydney, New South Wales, Australia; 3 Iron Metabolism Laboratory, QIMR Berghofer Medical Research Institute, Brisbane, Queensland, Australia; 4 School of Geosciences, University of Sydney, Sydney, New South Wales, Australia; 5 School of Chemistry and Molecular Bioscience, University of Queensland, Brisbane, Queensland, Australia; Lady Davis Institute for Medical Research, CANADA

## Abstract

Hephaestin is a large membrane-anchored multicopper ferroxidase involved in mammalian iron metabolism. Newly absorbed dietary iron is exported across the enterocyte basolateral membrane by the ferrous iron transporter ferroportin, but hephaestin increases the efficiency of this process by oxidizing the transported iron to its ferric form and promoting its release from ferroportin. Deletion or mutation of the hephaestin gene leads to systemic anemia with iron accumulation in the intestinal epithelium. The crystal structure of human ceruloplasmin, another multicopper ferroxidase with 50% sequence identity to hephaestin, has provided a framework for comparative analysis and modelling. However, detailed structural information for hephaestin is still absent, leaving questions relating to metal coordination and binding sites unanswered. To obtain structural information for hephaestin, a reliable protocol for large-scale purification is required. Here, we present an expression and purification protocol of soluble mouse hephaestin, yielding milligram amounts of enzymatically active, purified protein using the baculovirus/insect cell system.

## Introduction

**Fig 1:**
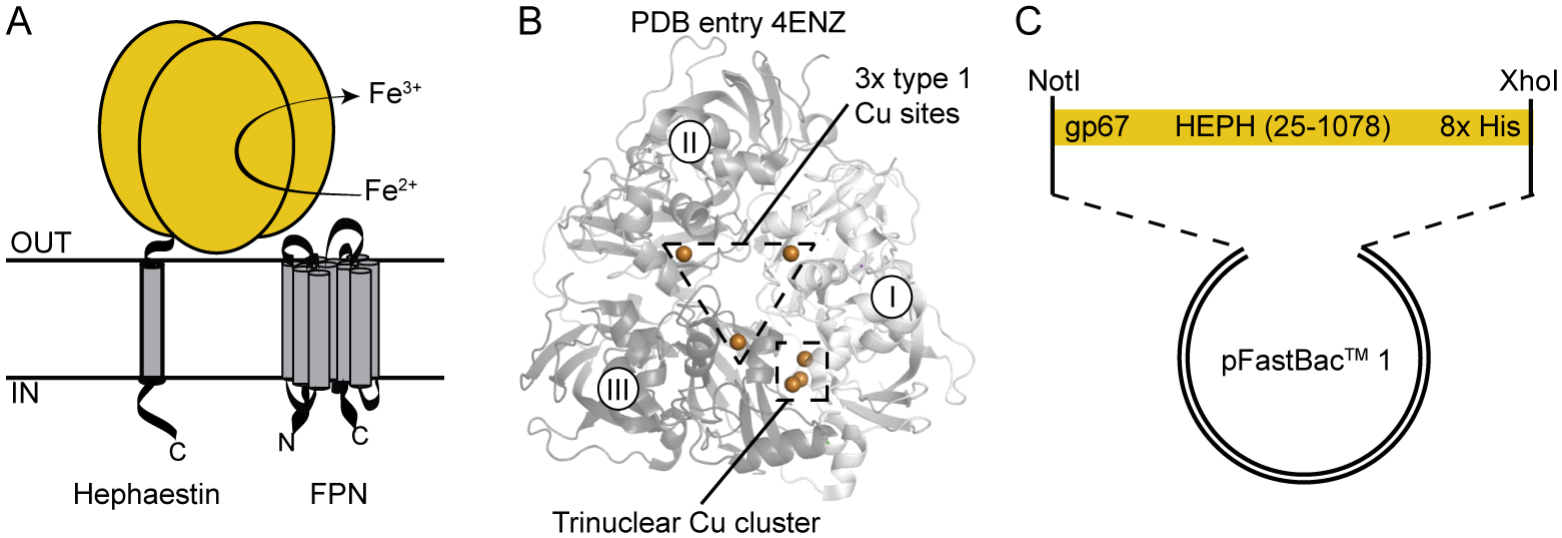
Hephaestin architecture and construct design. **A**, Schematic illustration of HEPH function. Ferroportin (FPN) exports divalent iron into plasma, where HEPH catalyses the conversion to trivalent metal. **B**, The structure of ceruloplasmin shown in cartoon representation, with the three copper binding domains coloured in different shades of grey. Copper ions are shown as orange spheres. In ceruloplasmin, 3 peripheral Type I copper sites shuttle electrons from the Fe^2+^—Fe^3+^ oxidation, with the electrons being transferred to the trinuclear copper cluster. **C**, The overall construct design used in this study.

Iron is a critical element for many cellular processes. However, iron is also toxic in high concentrations due to its ability to catalyze reactions leading to the generation of reactive oxygen species, requiring iron homeostasis to be tightly regulated. In mammals, dietary iron is absorbed through the apical membrane of enterocytes via the membrane transporter divalent metal-ion transporter 1 (DMT1). It is subsequently either stored in intracellular ferritin, or transported across the basolateral membrane of enterocytes (and into the circulation) via the ferrous iron exporter ferroportin (FPN). Iron transported through FPN is not released efficiently into the plasma in the absence of a multicopper ferroxidase [1,2], which oxidizes Fe^2+^ to Fe^3+^. Hephaestin (HEPH), a membrane anchored ferroxidase with a large ~120kDa ectodomain, is the main ferroxidase associated with the transport activity of FPN in the small intestine (Fig 1A). The physiological importance of HEPH-catalyzed ferroxidation is illustrated by HEPH in-frame deletion mice (*sla* mouse, expressing a truncated form of HEPH), in which iron export from the intestinal epithelium to plasma is significantly impaired [3]. This impairment causes anemia and iron accumulation in duodenal enterocytes. Recent studies of *Heph* knockout mice have also shown iron accumulation in the brain, and studies with *Heph*/*Cp* double knockout mice have implicated HEPH in maintaining iron homeostasis in some other tissues [4,5]. Iron accumulation and changes in iron distribution in regions of the brain are key features of both aging and neurodegenerative diseases, such as Alzheimer’s disease, Parkinson’s disease, and multiple sclerosis [6–11].

HEPH shares approximately 50% sequence identity with the soluble plasma multicopper ferroxidase ceruloplasmin. The crystal structure of human ceruloplasmin was previously determined, revealing the presence of six copper ions, 3 Type I peripheral centers and one catalytic trinuclear cluster (Fig 1B) [12]. The Type I sites accept electrons from the oxidation of substrate (Fe^2+^ to Fe^3+^), and the electrons from four substrate equivalents are transferred to the trinuclear cluster where they are coupled to the four-electron reduction of O_2_ to H_2_O [13]. Crystal soaking experiments further detailed two ferrous iron binding sites, each in the proximity of one of the Type I copper centers [14]. Comparative structural modeling has indicated an analogous architecture for HEPH [15]. However, preparations of recombinant human HEPH expressed in *Pichia pastoris* or baby hamster kidney (BHK) cells have indicated between 3 and 4 copper ions per HEPH molecule [14,16,17]. This discrepancy could indicate that the recombinant production of HEPH leads to incomplete copper loading, or, that HEPH has a different architecture than ceruloplasmin. In addition, the nature and number of the iron binding sites is not clear. Previous mutagenesis studies have indicated that only one of three predicted iron binding sites coordinates iron, possibly due to unorthodox sequence composition of the predicted binding sites [14].

In seeking to resolve these and other fundamental questions about HEPH, a crystal structure would be highly desirable. For this, a first step is to develop a reliable and cost-effective protocol for large-scale expression and purification. Previous HEPH purification protocols have led to either a lower copper content (BHK cells; 3.13 Cu atoms/protein molecule) or lower yield (*Pichia*; 50–75 μg/L culture) than anticipated. Here we report a protocol using the baculovirus and insect cell expression system to producing milligram quantities of secreted mouse HEPH, which shares 85% sequence identity with the human protein. Our purification protocol produces protein of >95% purity which appears stable for weeks at 4°C. Using inductively coupled plasma mass spectrometry (ICP-MS), we measured the copper content of the purified protein at an average of 4.04 atoms per protein molecule.

## Materials and methods

### Materials

Oligonucleotides were from IDT DNA (San Diego, USA). Phusion High-Fidelity DNA polymerase, enzymes for DNA manipulation, and endoglycosidases were from New England Biolabs (Beverly, USA). All polymerase chain reaction (PCR) and cloning steps were performed using established protocols [18]. Bac-to-Bac Baculovirus Expression System and antibodies were purchased from Thermo Fisher Scientific. Insect-XPRESS^™^ Protein-free Insect Cell Medium with L-glutamine was from Lonza (Switzerland). Ni-NTA resin was purchased from Qiagen, and polyprep columns were from Bio-Rad. All other reagents were purchased from Sigma–Aldrich, unless otherwise noted.

### Animals and tissue collection

C57BL/6 mice (WT) were obtained from the Animal Resource Centre (Perth, Australia). Ceruloplasmin knockout (CpKO) mice were originally generated by Dr. Leah Harris from Washington University (St. Louis, MO, USA) [19]. The strain located at QIMR Berghofer was obtained from Dr. Joshua Dunaief at the University of Pennsylvania, and was maintained on the C57BL/6 background. The mice had unlimited access to a standard rodent diet (Norco Stockfeeds, South Lismore, NSW, Australia) and water. Mice were euthanized by CO_2_ exposure and blood was drawn from the inferior vena cava. Fresh blood samples were immediately transferred to clean microcentrifuge tubes and allowed to clot at room temperature to obtain serum. All animal studies were approved by the QIMR Berghofer Animal Ethics Committee.

### Construct design and expression of truncated HEPH

The coding sequence for the soluble HEPH domain (amino acid residues 25–1078), excluding both the 5′ region encoding the intrinsic secretion signal peptide (residues 1–24) and the 3′ region coding for the membrane anchoring transmembrane helix, was subcloned from a vector containing the full-length mouse HEPH (gene Accession No NM_001159628). In the first PCR reaction, a gene-specific forward primer was used (primer I, GCGCATTCTGCCTTTGCG ATACCAACTGATGGGGCCATTC; underlined sequence corresponds to the gp67 overlapping region) whilst the reverse primer incorporated a C-terminal 8x His tag, to facilitate downstream purification, and an *XhoI* restriction site for subcloning (primer II, TCTAGACTCGAGTTA GTGATGATGATGGTGATGGTGATGGGTCATAGTGCTGAAATGTTC). A second PCR reaction amplified the secretion signal sequence from the gp67 baculovirus envelope glycoprotein [20] from an in-house vector, with the forward primer incorporating a *NotI* site at the 5′ end (primer III, TCTTTTTGCGGCCGCATGCTACTAGTAAATCAGTC) and the gp67 specific reverse primer (primer IV, CGCAAAGGCAGAATGCGCCG). The two PCR fragments were joined into one reading frame by overlap extension PCR using primers II and III as the reverse and forward primer respectively. The natural secretion signal peptide was replaced with that of the gp67 peptide as the natural peptide has previously proved unable to direct secretion of the recombinant HEPH in BHK cells [16].

The gp67-Heph-His fusion construct was subcloned into *NotI*/*XhoI*-digested baculovirus transfer vector pFastBac1 (ThermoFisher Scientific) and sequence verified. The virus was subsequently propagated using the Bac-to-Bac Baculovirus Expression System according to the manufacturer’s protocol. Briefly, the resulting vector carrying the gp67-Heph-His gene was transformed into *E*. *coli* DH10Bac cells containing the baculovirus genome (bacmid DNA). Transposition occurred between pFastBac1 and the bacmid to generate a recombinant bacmid with gp67-Heph-His gene. Positive clones were selected and the recombinant bacmid was isolated and transfected into *Spodoptera frugiperda* (*Sf*9) cells [21] for propagation of recombinant baculovirus.

### Expression of HEPH in insect cells

For large scale expression of secreted mouse HEPH protein, *Sf*9 or *Trichoplusia ni* BTI-TN-5B1-4 (High Five) cells [22,23] were infected at 2 × 10^6^ cells/mL with P3 virus at an optimal multiplicity of infection (MOI) of 2.5 in Insect-XPRESS^™^ Protein-free Insect Cell Medium with L-glutamine and incubated at 27°C with shaking at 130 rpm. The growth medium was supplemented with 25 μM copper sulfate as the intracellular copper concentration has been shown to have a dramatic effect on the turnover rate of HEPH [24]. Forty-eight hours post-infection, the medium containing the recombinant secreted protein was clarified by two centrifugation steps: (i) to remove cells, the medium was centrifuged at 500 ×*g* for 10 minutes, after which; (ii) the medium was further clarified by centrifugation at 20,000 ×*g* for 20 minutes to remove smaller cell debris. The protein was purified from the clarified medium within 24 hrs of collection.

### Purification of secreted HEPH

The HEPH protein was initially purified using a two-step protocol, with all steps carried out at 4°C. The secreted His-tagged HEPH was first purified by adding 4 mL Ni-NTA resin, pre-equilibrated with buffer 1 (10 mM Tris, pH 8 and 300 mM NaCl) to the clarified medium and subsequently incubated for 1 hr under stirring. Following this, the resin was loaded onto a polyprep gravity flow column. The resin was washed with 20 column volumes (80 mL) of buffer containing 10 mM imidazole pH 8, 300 mM NaCl and 10% glycerol (buffer 2). The bound protein was eluted with 15 mL of elution buffer (200 mM imidazole, pH 8, 300 mM NaCl and 10% glycerol).

The eluted protein was buffer exchanged into 20 mM Tris, pH 8 and 100 mM NaCl by repeated (2x) concentration/dilution steps using an Amicon Ultra-15 centrifugal filter unit (50 kDa cutoff; Millipore), and finally concentrated to 1.8 mL. The concentrated protein was loaded onto a HiLoad 16/600 Superdex^™^ 200 prep grade size exclusion column (GE Healthcare), pre-equilibrated with buffer containing 20 mM Tris pH 8, and 100mM NaCl. Peak fractions were analysed by SDS–PAGE (4–12% Bis–Tris gel; Invitrogen).

Given the high purity of HEPH, we subsequently altered the protocol to a one-step ‘batch’ protocol. After incubation with Ni-NTA resin, the sample was centrifuged at 500 × *g* for 5 minutes. The supernatant was discarded, and the Ni-NTA pellet was resuspended in a small volume of buffer 1 and applied to a gravity flow polyprep column. The sample was subsequently washed and eluted as above. The protein concentration was determined using a theoretical molar extinction coefficient of 189385 M^-1^cm^-1^ for the absorbance at 280 nm. Eluted protein was concentrated to 10–20 mg/mL and stored at -80°C until further use.

### Western blot analysis

In order to further confirm the identity of purified HEPH, we used both a mouse antibody directed against the C-terminal 8x His tag of the HEPH construct and an in-house generated rabbit anti-HEPH antibody [25]. Briefly 0.5 μg of HEPH protein samples (with and without copper) were resolved by electrophoresis on Novex^™^ 4–12% Tris-Glycine gels (ThermoFisher Scientific), followed by transfer to PVDF membranes (Millipore, MA) and blotting with primary mouse anti-His Ab (ThermoFisher Scientific) or rabbit anti-HEPH Ab. HRP-conjugated rabbit anti-mouse or goat anti-rabbit Abs were used as secondary antibodies, respectively. Chemiluminescent images were obtained using a ChemiDoc^™^ imaging system (Bio-Rad).

### Endoglycosidase treatment of purified HEPH

The presence of glycosylation on the purified protein sample(s) was analysed using the endoglycosidases PNGase A and PNGase F. Samples were treated with the enzymes in a 10:1 ratio under denaturing reaction conditions according to the manufacturer’s protocol. To assess the presence of glycosylation, the samples were subsequently analysed on SDS-PAGE gels to observe any mobility shift.

### Oxidase activity assays

*p*-Phenylenediamine (*p*PD) (Sigma-Aldrich, cat # P6001) and (NH_4_)_2_Fe(SO_4_)_2_ (Sigma-Aldrich, cat # 215406) were used as substrates to determine the ferroxidase activity in *p*PD and ferrozine (Fz, Sigma-Aldrich, cat # 160601) assays, respectively. The principles and protocols for both assays have been described in detail previously [26,27]. However, the results obtained in the current study (with the exception of the kinetic measurments) were based on a slightly modified approach, which uses a 96-well plate-based format. Absorbance was measured using BioTek^®^ spectrophotometers (Synergy H4 or Powerwave, BioTek Instruments, Inc., Australia).

#### *p*PD assay

Briefly, the reaction mix consisted of 500 μg serum proteins (from WT or CpKO mice), or 15, 50 or 100 μg HEPH, with or without inhibitors, in 0.125 M sodium acetate buffer, pH 5.0. The assay blank contained only buffer and water (in place of the sample). The mix was then aliquoted into 96-well plates in triplicate (180 μL/well). For inhibition studies, 100 μM D-penicillamine, a copper-specific chelator was added to the reaction mix and incubated for ~1 hour at RT with gentle mixing, prior to the addition of substrate. To initiate the reactions, 20 μL of freshly-made 1% *p*PD solution was added to each well and the plate was incubated at 37°C in the dark. The absorbance at 530 nm was recorded over 150 min at 15 min intervals.

#### Fz assay

The reaction mix composition, assay blank and inhibitor study conditions for the Fz assay were the same as for the *p*PD assay, with the exception of the substrate. The reaction was initiated by adding (NH_4_)_2_Fe(SO_4_)_2_ solution to the reaction mix to achieve a final concentration of 50 μM. Every 15 min for 1 hr, 200 μL aliquots of the reaction mixtures were added to the wells of 96-well plates that had been preloaded with 20 μL of 30 mM Fz solution. At the end of the experiment, the absorbance at 570 nm was recorded.

#### Kinetic measurements of HEPH activity

The enzyme kinetics of the HEPH were studied using a Fz-based assay, with Fe^2+^ as a substrate. The reaction mix consisted of 0.125 M sodium acetate buffer, pH 5.0 and (NH_4_)_2_Fe(SO_4_)_2_ at various concentrations (0, 2.5, 5, 7.5, 15, 25, or 50 μM). The enzymatic oxidation of Fe^2+^ to Fe^3+^ was initiated by adding 0.2 μM HEPH into the reaction mix. At 0, 1, and 2 min, a portion of the reaction mix was removed and added to acrylic cuvettes (1 cm pathlength; Sarstedt) that had been preloaded with 30mM Fz solution to deplete the substrate so that the reaction was stopped. The absorbance at 562nm was obtained using a BioMate 3 spectrophotometer (ThermoFisher Scientific) and the Fe^2+^ concentration was determined using Beer’s law and a molar extinction coefficient for the Fz-Fe^2+^ complex of ε_562_ = 27900 M^-1^cm^-1^ [28]. All reactions were corrected for autoxidation of Fe^2+^. The initial velocity of the reaction was plotted against the substrate concentration using GraphPad Prism software (V7.02, San Diego, CA, USA) and the Michaelis-Menten nonlinear regression curve fitting was performed to determine the *K*_m_ and *V*_max_.

### Copper content analysis using ICP-MS

To assess the presence of copper, UV/visible absorbance spectra were recorded from 300 nm to 800 nm using the FLUOstar^®^ Omega microplate reader. The copper content of the purified sample was determined using ICP-MS according to manufacturer’s protocols (Perkin Elmer) as previously reported [29]. In brief, 75 mL of purified protein (12.83 mg/mL) was dissolved in 5 mL of high purity concentrated nitric acid (HNO_3_) and microwave digested for 30 min at 160°C to obtain complete digestion. The extract was further diluted to 200 mL and subsequently analysed for copper content. Measurements were made using a Perkin Elmer Nexion 300XX ICP-MS instrument. The instrument was calibrated using 10 and 25 parts per billion (ppb) of copper prepared in 1% (v/v) nitric acid. Results were expressed as micromole per liter concentrations of metal (μmol/L). The concentration of Cu was calculated as μg of metal per mg of protein based on the protein concentration.

## Results & discussion

HEPH is a critical component of mammalian iron homeostasis, yet important questions regarding its copper content and iron binding sites remain. Previous structural studies of ceruloplasmin have provided a framework for the structural architecture of HEPH through modelling on the basis of their sequence similarity [15]. However, previous recombinant HEPH purification protocols have generated protein with consistently lower than expected copper ion content. Whilst protein production by BHK cells provided sufficient yields, the copper content was the lowest [16]. Conversely, expression and purification in *Pichia* had the highest copper content, but the yield was low and the protein generated appeared to be unstable in solution [14,17]. In an attempt to merge the aspects of ‘high’ copper content, high yield, and protein stability, we generated a protocol for mouse HEPH expression using the baculovirus and insect cell system.

### Construct design and expression of HEPH

**Fig 2:**
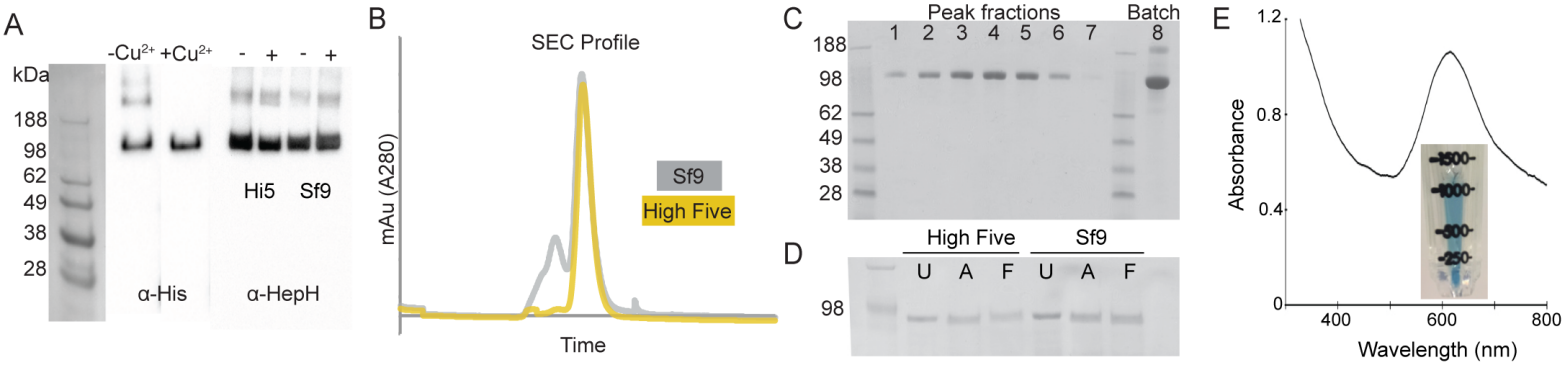
Purification of recombinant mouse HEPH. **A**, Western blot of purified samples using antibodies directed against the C-terminal His-tag (left) and HEPH (right). Adding copper sulfate to the culture media made no significant difference when analysed by Western blotting. **B**, Size exclusion chromatograms of protein purified from *Sf*9 and High Five cells. **C**, SDS-PAGE of peak fractions (lane 1–7) from size exclusion chromatography. Lane 8 is concentrated protein from batch purification. **D**, SDS-PAGE analysis of endoglycosidase treatment of purified HEPH protein from High Five and *Sf*9 cell expression. U = untreated, A = PNGase A, F = PNGase F. **E**, Concentrated protein had a distinct blue colour from the copper content of the protein, which had an absorbance peak at 611 nm.

Early expression trials of mouse HEPH using a construct that included the native secretion signal peptide indicated only partial secretion. This is likely due to heterologous signal peptides being poorly recognised by the insect cell translation machinery [30]. To circumvent this, we generated an expression construct for the production of HEPH incorporating an N-terminal gp67 secretion signal peptide and a C-terminal His-tag in the pFastBac system (Fig 1C). The baculoviral glycoprotein gp67 is the most abundant envelope surface glycoprotein and is essential for the entry of baculovirus particles into host insect cells. The gp67 signal peptide mediates the forced secretion of the recombinant protein, even if it is normally not secreted [20,30,31]. During transport across the cell membrane, the signal peptide is cleaved and the recombinant protein can be purified from the supernatant of infected cell cultures. The construct generated was verified by *NotI*/*XhoI* digestion and sequencing. This construct yielded secreted protein and the protein identity was confirmed by Western blotting using antibodies directed against the C-terminal His-tag and HEPH itself (Fig 2A).

To optimise the expression levels, we trialled expression in both *Sf*9 [21] and High Five [22,23] cells. The latter are purported to provide higher yields for secreted and glycosylated proteins [32,33]. For both cell lines, harvesting 48 hours post infection gave the highest yield of expressed and secreted protein.

### Purification of secreted mouse HEPH

For large-scale protein expression, 4 L of cells were infected at a MOI of ~2.5. As the resulting protein was secreted into the medium, remaining cells and cell debris were clarified by centrifugation prior to binding the His-tagged protein to Ni-NTA resin. Whilst there was no observable difference between the cell lines in terms of overall yield (1–2 mg/L), size exclusion chromatography (SEC) of the concentrated protein from High Five cells gave a more monodisperse peak (Fig 2B). The final protein purity from High Five cell expression is estimated to be >99% (Fig 2C).

It has previously been shown that the two cell lines have differences in how they glycosylate proteins, with the High Five cells being capable of α-1,3-core fucosylation, which is not observed in *Sf*9 cells [34,35]. Hence, to explore any potential glycosylation of the samples, we treated the protein with the endoglycosidases PNGase F and PNGase A. The former is an amidase that cleaves between the innermost GlcNAc and asparagine residues of high mannose, hybrid, and complex oligosaccharides from *N*-linked glycoproteins. PNGase A differs from PNGase F in that it cleaves *N*-linked glycans, with or without α-1,3-core linked fucose residues. The protein samples were treated with the endoglycosidases according to the manufacturer’s protocols under non-denaturing conditions and the samples were subsequently analysed on SDS-PAGE to observe any mobility shift (Fig 2D). However, no shift was observed, indicating that the recombinant protein is not glycosylated.

To increase the recovery, yield, and purification speed, we also altered the protocol to perform the initial step in ‘batch’. This was done by collecting the Ni-NTA incubated with the clarified media using low-speed centrifugation. This rapid one-step purification yielded a highly pure (>95%) protein. Following elution, the protein was concentrated to 10–20 mg/mL and stored frozen until required. The overall yield of purified HEPH from the ‘batch’ method was approximately 2 mg/L of culture.

### Copper content of purified mouse HEPH

The multicopper ferroxidases require bound copper ions for their catalytic activity. Copper deficiency in mice has accordingly been shown to dramatically reduce HEPH protein levels and ferroxidase activity [36]. The purified mouse HEPH protein from our protocol had a distinct blue colour and exhibited an absorption maximum at 611 nm, which is characteristic of proteins with a Type I copper site (Fig 2E). To analyse the specific copper content of the purified protein, we digested the protein to release bound copper ions and subsequently used inductively coupled plasma mass spectrometry (ICP-MS) to measure the copper concentration. The purified HEPH had an average of 4.04 copper atoms per protein molecule. Compared with previously published HEPH purification protocols, our sample has a higher copper content than that of protein produced in BHK cells [16], and, whilst it is similar to the copper content of protein generated in *Pichia* [14,17], the yield is much higher (50–75 μ /L vs. 1–2 mg/L).

Curiously, the copper content in recombinant HEPH has consistently been shown to be lower than predicted. As noted in previous studies, this could be due to incomplete copper loading of the expressed protein, or HEPH could have an intrinsically lower copper content than ceruloplasmin [14]. Assuming an overall similar structure and mechanism to ceruloplasmin and other multicopper oxidases, a minimum of 4 copper ions would be required for activity, with one Type I copper site transferring electrons from the oxidation of Fe^2+^ to the catalytic trinuclear cluster. It is thus theoretically possible that HEPH only contains 4 intrinsic copper ions, which would correspond with the copper content of the recombinant preparations from both *Pichia* and the protein described here. Supporting this, mutagenesis of the predicted substrate (iron) binding sites in the human HEPH protein—each in the proximity of one of the three predicted Type I copper sites—has indicated that only one site is functionally important [14], whilst crystal soaking experiments have revealed that at least two sites are present in ceruloplasmin [37]. In addition, although being a smaller protein and missing one of the copper containing domains of mammalian ferroxidases, the Fet3p protein from yeast provides a functional example of a ferroxidase with only 4 intrinsic copper ions [38].

### Oxidase activity and enzyme kinetic characterization of HEPH

**Fig 3:**
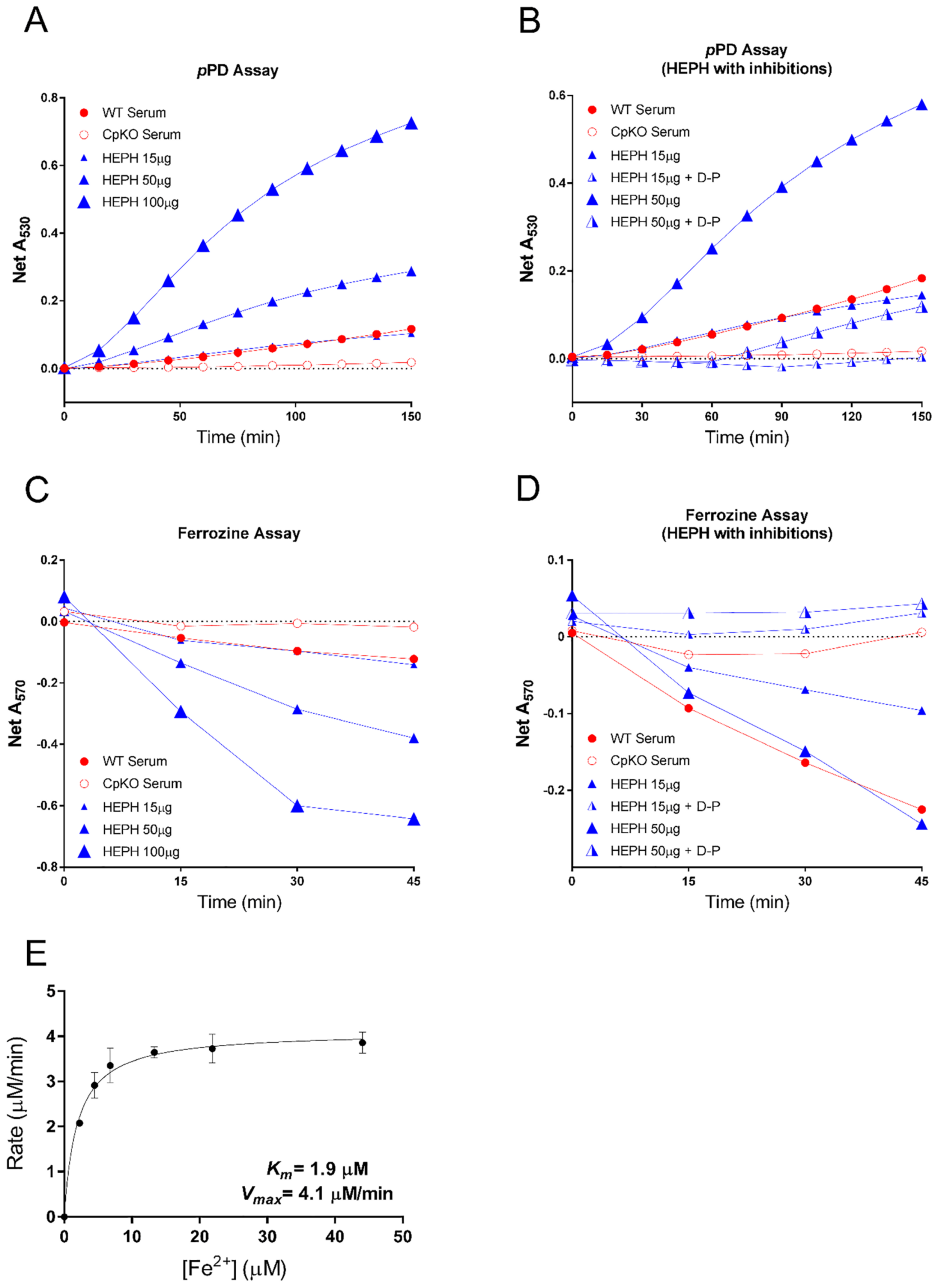
Oxidase activity of mouse HEPH. In all assays WT mouse serum and serum from a mouse CpKO were used as control. Details can be found in the main text. **A**, An oxidase assay monitoring the oxidation of *p*PD (increase of A_530_). The rate of HEPH catalysed oxidation reaction increased with an increasing protein concentration. **B**, *p*PD oxidation by HEPH was inhibited by the copper-specific chelator D-penicillamine (D-P). **C**, A ferrozine-based assay was also used to follow the oxidation of Fe^2+^ to Fe^3+^ by HEPH (monitored as a decrease of A_570_). Similarly to the *p*PD assay, an increased catalytic activity can be observed with an increasing amount of HEPH present. **D**, The catalytic activity was, as in the pPD assay, inhibited by addition of D-P. **E**, A velocity versus substrate concentration curve used to obtain *K*_m_ and *V*_max_ for HEPH. Error bars represents 1 S.D, N = 3.

The oxidase activity of purified HEPH was established using both *p*PD- and Fz-based assays where serum proteins from WT and CpKO mice were used as controls. In the *p*PD assay, the absorbance monitored at 530 nm showed the rate of oxidation of *p*PD. Here, 15 μ5 purified HEPH had a similar activity to that of 500 μ5 WT mouse serum, whereas increasing concentrations of HEPH yielded in higher activity (Fig 3A). In contrast, the addition of D-penicillamine, a copper-specific chelator, led to a drastic decrease in the oxidation of pPD (Fig 3B). In the Fz assay, the oxidation of Fe^2+^ to Fe^3+^ was monitored as a decrease of the Fz-Fe^2+^ complex, which absorbs at 570 nm. Hence, in this assay, increasing concentrations of HEPH illustrated higher activity in the form of a greater decrease in A_570_, indicative of the conversion of Fe^2+^ to Fe^3+^ (Fig 3C). Similarly to the *p*PD assay, addition of D-penicillamine successfully inhibited this activity (Fig 3D).

As these assays clearly demonstrated the protein to be active and responsive to inhibitors, we also used a Fz-based oxidase assay to determine the *K*_m_ and *V*_max_ of the purified protein (Fig 3E). The *K*_m_ for Fe^2+^ was determined to be 1.9 μM, which is similar to that of Fet3p (2.0–4.8 μM) [39,40], HEPH purified from BHK (2.1 μM) [16] and *Pichia* cells (3.2 μM) [17]. However, our protein has significantly higher *V*_max_ than the BHK and *Pichia* produced HEPH protein (4.1 μM min^-1^ versus 0.5 and 0.15 μM min^-1^, respectively). Compared to the BHK produced protein, the improved catalytic rate is likely a result of the higher copper content of the purified HEPH protein. With an average of 4 copper ions, the protein is expected to have both a catalytic trinuclear copper site and a peripheral Type I site in place, similar to the Fet3p protein. Indeed, the *V*_max_ of the purified HEPH protein is more similar to that of Fet3p (1.0–1.8 μM min^-1^). However, despite the significant improvements made in protein production with higher copper content and activity, further studies of HEPH are required to provide definitive data on its intrinsic copper content.

## Conclusions

We have generated an expression and purification protocol for mouse HEPH which yields milligram quantities of pure and monodisperse protein. The copper content of the protein is similar to a previously published expression protocol in *Pichia*, but our new protocol provides a much higher yield and protein with improved kinetic properties. This high-yield and active ‘high’ copper content protein constitutes a platform for future structural studies.
